# Anacardic Acid Reduces Acetylation of H4K12 in Mouse Oocytes during Vitrification

**DOI:** 10.22074/cellj.2019.5601

**Published:** 2018-08-07

**Authors:** Alaleh Ghazifard, Mohammad Salehi, Marefat Ghaffari Novin, Mojgan Bandehpour, Somayeh Keshavarzi, Vahid Fallah Omrani, Maryam Dehghani-Mohammadabadi, Reza Masteri Farahani, Ahmad Hosseini

**Affiliations:** 1 Department of Reproductive Biology and Anatomical Sciences, Faculty of Medicine, Shahid Beheshti University of Medical Sciences, Tehran, Iran; 2 Cellular and Molecular Biology Research Center, Shahid Beheshti University of Medical Sciences, Tehran, Iran; 3 Department of Biotechnology, School of Advanced Technologies in Medicine, Shahid Beheshti University of Medical Sciences, Tehran, Iran; 4 Department of Transgenic Animal Science, Stem Cell Technology Research Center, Tehran, Iran

**Keywords:** Acetylation, Anacardic Acid, Histone, Oocyte, Vitrification Cell Journal(Yakhteh), Vol 20, No 4, Jan-Mar (Winter) 2019, Pages: 552-558

## Abstract

**Objective:**

Over the last years, vitrification has been widely used for oocyte cryopreservation, in animals and humans; however, it frequently causes minor and major epigenetic modifications. The effect of oocyte vitrification on levels of acetylation of histone H4 at lysine 12 (AcH4K12), and histone acetyltransferase (*Hat*) expression, was previously assessed; however, little is known about the inhibition of *Hat* expression during oocyte vitrification. This study evaluated the effect of anacardic acid (AA) as a Hat inhibitor on vitrified mouse oocytes.

**Materials and Methods:**

In this experimental study, 248 mouse oocytes at metaphase II (MII) stage were divided in three experimental groups namely, fresh control oocytes (which were not affected by vitrification), frozen/thawed oocytes (vitrified) and frozen/thawed oocytes pre-treated with AA (treatment). Out of 248 oocytes, 173 oocytes were selected and from them, 84 oocytes were vitrified without AA (vitrified group) and 89 oocytes were pretreated with AA, and then vitrified (treatment group). Fresh MII mouse oocytes were used as control group. *Hat* expression and AcH4K12 levels were assessed by using real-time quantitative polymerase chain reaction (PCR) and immunofluoresce staining, respectively. In addition, survival rate was determined in vitrified and treatment oocytes.

**Results:**

*Hat* expression and AcH4K12 modification significantly increased [4.17 ± 1.27 (P≤0.001) and 97.57 ± 6.30 (P<0.001), respectively] in oocytes that were vitrified, compared to the fresh oocytes. After treatment with AA, the Hat mRNA expression and subsequently H4K12 acetylation levels were significantly reduced [0.12 ± 0.03 (P≤0.001) and 89.79 ± 3.20 (P≤0.05), respectively] in comparison to the vitrified group. However, the survival rate was not significantly different between the vitrified (90.47%) and treatment (91.01%) groups (P>0.05).

**Conclusion:**

The present study suggests that AA reduces vitrification risks caused by epigenetic modifications, but does not affect the quality of vitrification. In fact, AA as a Hat inhibitor was effective in reducing the acetylation levels of H4K12.

## Introduction

Preservation of embryos and gametes is one of the main concerns of assisted reproductive technology (ART). In most cases, oocytes should to be stored, for future fertilization, based on lifestyle or medical conditions (1, 2). Currently, vitrification is a gold-standard approach for cryopreservation of oocytes or embryos (3). 

The effects of cryoprotectants on gametes and embryos are considerable and could be categorized into early and late onset types. In early onset, reducing the viability of gametes, and the effect of fertilization functions, lead to impairments of embryo formation. This effect can be measured by counting embryo formation. Late onset effects that will emerge after birth include changes in genetics and epigenetics (4). Epigenetic modifications- with global effects on genome and delayed subsequent effects on the embryo-are more important in clinical settings (5). 

Different types of epigenetic modifications affect gene expression, through direct DNA modifications or via modification of DNA-associated proteins. DNA methylation and demethylation directly affect DNA while acetylation, phosphorylation, methylation and ubiquitination affect histones, as the main DNA-associated proteins (6). It has been demonstrated that several lysine residues on histones H3 and H4 remain deacetylated during oocyte meiosis, but they become acetylated in preimplantation embryos (7, 8).

Histone acetyltransferases (Hats) and histone deacetylases (Hdacs) are enzymes that play significant roles in regulation of genes expression. Investigations have demonstrated that regulation of acetylation balance is vital for cell function (9). Several cell functions, such as chromosome decondensation, DNA double-strand break repair, and transcription are intently associated with histone acetylation (10). 

Previous studies showed that vitrification affects histone acetylation, which leads to open chromatin and transcription activities. It was demonstrated that histone acetylation increases in the vitrified oocytes resulting in large epigenetic influences in oocytes and early embryos (11-14). Also, it was reported that vitrification procedure could increase the level of *Hat* expression and therefore enhance H4K12 acetylation level (13).

Several natural products have been shown to have Hat inhibitory properties. For example, anacardic acid (AA) as an inhibitor of Hats was used to design novel small molecule that can inhibit Hats (15). AA is found in the nutshell of Anacardium occidentale. This bioactive phytochemical has received great attention from pharmaceutical companies and chemobiology researchers (16). Nevertheless, this Hat inhibitor has not yet been applied to oocyte vitrification, and little is known about the inhibition of *Hat* expression during the vitrification of oocytes. 

The aim of the present study was to investigate the effect of AA as a Hat inhibitor in the process of vitrification of oocytes by mean of immunocytochemical staining and real-time quantitive polymerase chain reaction (PCR). 

## Materials and Methods

In this experimental study, all chemicals and media were obtained from Sigma-Aldrich (St. Louis, MO) unless otherwise mentioned. 

All the procedures were approved by the Ethics Committee of Shahid Beheshti University of Medical Sciences, Tehran, Iran. Mice were kept at 20-28°C with 12 hours/12 hours light/dark cycles and they had free access to food and water. 

### Oocyte collection

Female B6D2F1 mice (6 to 8 weeks old) were purchased from Pasteur Institute, Tehran, Iran and superovulated with intraperitoneal injection of 10 IU pregnant mare serum gonadotrophin (PMSG). After 48 hours, 10 IU of human chorionic gonadotropin (HCG) was administered. After 14 hours, cumulus-oocyte complexes were collected from the oviductal ampulla in flushing holding medium (FHM) with 4 mg/mL bovine serum albumin (BSA). To remove oocytes from cumulus cells, they were immediately put in medium containing hyaluronidase. 

Metaphase II (M..) oocytes with normal morphology, regular contours and light coloration were selected and stored in K+ modified simplex optimized medium (KSOM) at 37°C with 5% CO_2_ until vitrification time.

Then, oocytes at M.. stage were grouped into three groups namely, fresh control group, vitrified group and treatment group. 

### Vitrification of mouse oocytes 

Mouse MII oocytes were vitrified in a two-step procedure using the KITAZATO Vitrification KIT (Kitazato Biopharmaceuticals, Japan). In order to vitrify, the cryotop (Kitazato) was used as a carrier. The test was performed using a procedure reported by Kuwayama (17). First, oocytes were pretreated with equilibration solution (ES) consisting of 7.5 % (v/v) ethylene glycol (EG) and 7.5 % (v/v) dimethylsulfoxide (DMSO) for 9 minutes at room temperature, and then transferred to vitrification solution (VS) consisting of 15 % (v/v) EG, 15 % (v/v) DMSO and 0.5 M sucrose. After being washed 3 times in less than 60 seconds, four to six oocytes in minimal VS (<1 µL) were transferred onto the cryotop carrier. The cryotop was immersed in liquid nitrogen. Subsequently, a plastic cap was placed over the straw, prior to storage in liquid nitrogen.

### Treatment with anacardic acid in vitrification 

A 25-µM solution of AA was made in DMSO. Oocytes were preincubated with 25 µM AA in KSOM medium for 40 minutes, then vitrified in ES and VS with 25 µM AA as follows: at the first, oocytes were pretreated in ES consisting of 7.5 % (v/v) EG and 7.5 % (v/v) DMSO for 9 minutes at room temperature, and then put into VS consisting of 15 % (v/v) EG, 15 % (v/v) DMSO and 0.5 M sucrose. After washing in less than 60 seconds, oocytes were transferred to the cryotop carrier and stored in liquid nitrogen.

### Thawing of oocytes

Vitrified oocytes were kept in liquid nitrogen for two weeks. The oocytes were thawed using a four-step dilution procedure (Kitazato Biopharmaceuticals, Japan). In brief, the protective cap was removed from the cryotop containing the oocytes and immersed into warming solution (0.5 M sucrose at 37°C) for 1 minute. After that, the oocytes were placed in diluent solutions containing 0.25 M sucrose for 3 minutes and 0.125 M sucrose for 5 minutes.

Then, the oocytes were placed in sucrose-free washing solution for 1 minute.

Oocytes were transferred into KSOM medium droplets after warming and then incubated at 37°C with 5% CO2 for at least 1 hour. The survival rate of oocytes in vitrified and treatment groups was assessed after thawing. Afterward, oocytes were washed in FHM medium with 4 mg/mL BSA and then prepared for immunolabeling process. 

### AcH4K12 analysis of oocytes by immunocytochemical staining 

MII oocytes of all three groups were immunostained with antibody against AcH4K12 (Abcam, Cambridge, UK, 1:500 dilution). In each of the three groups, 23 oocytes were examined.

Immunolabeling process was performed as previously described (18, 19), with slight modifications. Samples were fixed for 30 minutes in 4% paraformaldehyde, and permeabilized using 0.2% Triton X-100 in phosphate buffered saline (PBS) for 1 hour at 4°C. The fixed oocytes were blocked in PBS containing 3% BSA for 1 hour at room temperature and incubated with primary antibody at 4°C, overnight. On the next day, after extensive washing in FHM containing 0.1% polyvinyl alcohol (PVA), the oocytes were labeled with secondary fluorescein isothiocyanate (FITC)-conjugated antibody (1:200 dilution) for 1 hour at 37°C. Finally, the samples were washed using FHM containing 0.1% PVA and nuclear status of oocytes was evaluated by staining with 4’,6-diamidino-2-phenylindole (DAPI, 10 µg/mL) for 1 minutes at room temperature in the dark. Samples were mounted on slides after extensive washing. The fluorescence was monitored by an epifluorescence microscope (Nikon, Japan) with UV filters (371 nm). In order to study H4K12 acetylation, the photographs were analyzed by using ImageJ software (Version 1.45S, National Institutes of Health, USA). In negative control group, cells were not incubated with the primary antibody.

### Concurrent RNA extraction and cDNA synthesis 

Oocytes of all three groups (i.e. control, vitrified, and treatment groups) were placed in Eppendorf tubes containing 1.5 µL lysis buffer (20). Both Reverse Transcription (RT) and PCR were performed on an applied thermocycler (Bio-Rad, Hercules, CA). Afterwards, 2 µL random hexamer and 5 µL water were added to each 2 µL of oocyte sample, which were then transferred to the thermocycler for 5 minutes at 75°C. Next, the tubes were placed on ice, and 5X RT Buffer, 200 U/µl RT Enzyme, 10 mM dNTP, and 10 U/µl RNase inhibitor were added to the reaction mixture. 

In order to perform the reverse transcription step, the amplification program was followed at 25°C for 10 minutes, at 37°C for 15 minutes, at 42°C for 45 minutes and at 72°C for 10 minutes. After completion of the reverse transcriptase reaction, the samples were kept at 4°C, overnight; Then, to each sample PCR mixture containing 1.25 µL Taq Polymerase, 20.75 µL Master Mix 2 µL cDNA and 2 µL specific primer, was added (Table 1). 

The endogenous control, Hypoxanthine Phosphoribosyltransferase 1 (Hprt1), Hat1 and Hdac1 genes were used with initial denaturation at 94°C for 5 minutes followed by denaturation at 94°C for 15 seconds, annealing at 60°C for 30 seconds and extension at 72°C for 45 seconds repeated for 35 cycles. Final elongation step was performed at 72°C for 7 minutes. The mixture of 10 µL of PCR product with 1 mL loading buffer was electrophoresed on 2% agarose gel in TAE (Tris-acetate- EDTA) for 25 minutes. The products were visualized under shortwave UV.

### Real-time quantitative polymerase chain reaction

To assess the expression levels of *Hat1* and *Hdac1* genes, real-time quantitative PCR was performed by using Rotor-Gene Q instrument (QIAGEN). A total volume of 13 µL DNA Master SYBR Green I mix (Roche Applied Sciences) was used for real-time PCR according to the manufacturer’s instructions.

For each gene, 1 µM of primer was added (Table 1). The PCR procedure was done as follows: 5 seconds at 95°C, 3 minutes at 95°C for denaturation, 15 seconds at 60°C, 10 seconds at 72°C for amplification and 40 cycles of extension. The specificity of all individual amplification reactions was confirmed by melting curve analysis. *Hprt1* was used as the housekeeping gene. Three replications were performed and the expression of target mRNA was normalized against *Hprt1*.

### Statistical analysis

Data analyses were performed using SPSS Ver.20 (SPSS, Chicago, IL, USA). Means of fluorescent intensity of epigenetic markers were compared by nonparametric Mann- Whitney test. The relative levels of mRNA were analyzed by REST 2009 Software (QIAGEN). Data are expressed as means ± SD. P=0.05 and P=0.001 were considered significant and all experiment were performed at least in triplicates. 

**Table 1:** Primer sequences used in reverse transcription polymerase chain reaction (RT-PCR) and real time quantitative PCR


Entrez gene symbol	Gene name	Accession number	Primer sequence (5´-3^´^)	Product length (bp)

*Hat1*	Histone acetyltrasnferase1	NM_026115.4	F: TATGGCAATACAGGCACAGC	102
			R: TCAGCATCGCTCATGTCAG	
*Hdac1*	Histone deacetylases1	NM_008228.2	F: GGCATTGACGACGAATC	157
			R: TGGCGTGTCCTTTGATG	
*Hprt1*	Hypoxanthine Phosphoribosyltransferas1	NM_013556.2	F: TCCCAGCGTCGTGATTAG	138
			R: CGAGCAAGTCTTTCAGTCC	


## Results

A total of 248 MII oocytes were used in this study. The oocytes were categorized into three groups including fresh control oocytes, frozen/thawed oocytes (vitrified group), and frozen/thawed oocytes pre-treated with AA(treatment group). 

A total of 173 oocytes were subjected to vitrification. From 173 vitrified oocytes, 84 oocytes were vitrified without AA (vitrified) and 89 oocytes were pretreated by AA, and then vitrified (treatment). The survival rates after thawing in vitrified and treatment groups were 90.47% (76/84) vs. 91.01% (81/89), respectively. The survival rate was not significantly different between the vitrified and treatment groups (Table 2). 

**Table 2:** Effect of vitrification with and without pre-treatment with anacardic acid on oocyte survival


Group	Number of MII oocyte	Number of survival (%)

Control	75	-
Vitrified	84	76 (90.47)
Treatment	89	81 (91.01)


There was no significant difference between the vitrified and treatment groups (P>0.05).

### Vitrification affects H4K12 acetylation levels and Hat expression in oocytes

**Fig.1:**
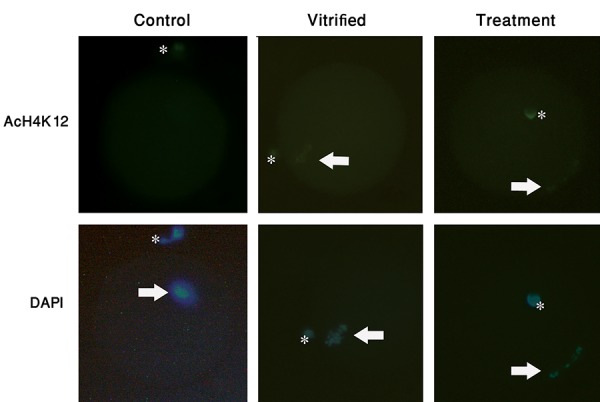
The acetylation levels of H4K12 in mouse MII oocytes in three groups namely, fresh control oocytes (that were not affected by vitrification), frozen/ thawed oocytes (vitrified cells) and frozen/thawed oocytes pre-treated with AA (treatment). In the treatment group, oocytes were preincubated with 25 µM AA for 40 minutes and then vitrified. Oocytes in the treatment group showed decreases in histone acetylation at H4K12. Oocytes were immunostained with an antibody specific for acetylated H4K12. The antibody was visualized using FITC-conjugated secondary antibody (green), and the DNA was stained with DAPI (blue). Asterisk indicates the polar body and arrow indicates the MII plate (scale bar: 20 µm).

Acetylation of histone H4 at lysine 12 was enhanced by vitrification, as confirmed by immunostaining with an antibody specific for acetylated H4K12 (Fig .1).

There was a significant increase in acetylation levels of H4K12 in vitrified oocytes compared to those of the fresh control (97.57 ± 6.30 vs. 8.57 ± 1.32, P<0.001) (Table 3). 

**Table 3:** Fluorescence intensity of acH4K12 during oocyte vitrification


Group	Number of MII oocyte	Fluorescence intensity (mean ± SD)

Control	23	8.57 ± 1.32^a^
Vitrified	23	97.57 ± 6.30^b, c^
Treatment	23	89.79 ± 3.20^b, d^


Different letters indicate significant differences between groups. ^a, b^; Significant differences between groups (P<0.001), and ^c, d^; Significant difference between vitrified and treatment groups (P=0.05).

**Fig.2:**
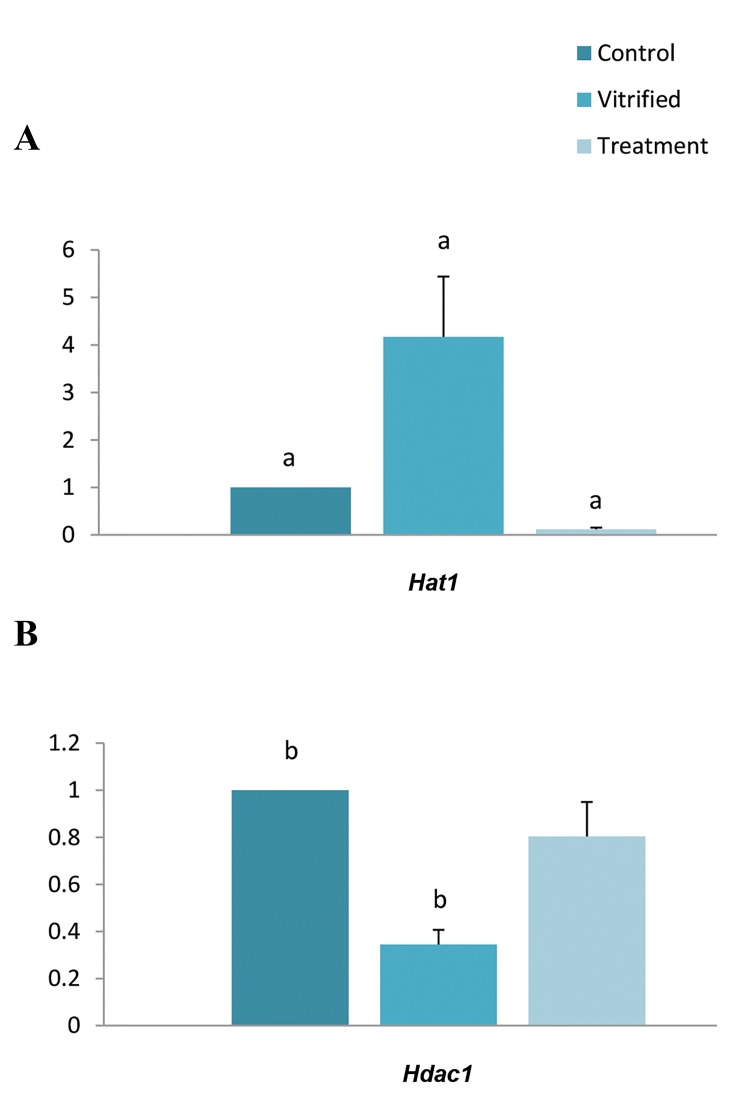
Relative expression levels of mouse *Hat1* and *Hdac1* genes in MII oocytes in the fresh control (that were not affected by vitrification), vitrified (frozen/thawed) and treatment (frozen/thawed cells pretreated with AA) groups. In the treatment group, oocytes were preincubated with 25 µM AA for 40 minutes and then vitrified. The mRNA levels of the genes were analyzed by qPCR and normalized against *Hprt1* mRNA level. A. The expression of *Hat1* mRNA was significantly elevated in the vitrified group compared to the fresh control group and a significant decrease in *Hat1* expression in AA-treated group was observed when compared to the vitrified group. B. The expression level of *Hdac1* was significantly reduced following vitrification. MII; Metaphase II, AA; Anacardic acid, ^a^; A significant difference between groups (P≤0.001), and ^b^; A significant difference between control and vitrified groups (P≤0.001).

The expression of *Hat1* mRNA was significantly elevated (4.17 ± 1.27, P=0.001) in the vitrified group without AA treatment compared to the fresh control group (Fig .2A) while the expression level of *Hdac1* was significantly reduced following vitrification (0.34 ± 0.06, P≤0.001) (Fig .2B). 

### AA reduced H4K12 acetylation levels and Hat expression in oocytes during vitrification

In this experiment, the acetylation of H4K12 was altered by *Hat1* inhibitors. AA-treated oocytes indicated a significant decrease in the fluorescence signal as compared to the vitrified oocytes (without AA, Fig .1). AcH4K12 levels in AA-treated group (89.79 ± 3.20) was significantly lower than those of vitrified group (97.57 ± 6.30) (P=0.05, Table 3).

The relative expression level of *Hat1* was decreased significantly by AA treatment (P=0.001). A significant decrease in *Hat1* expression in AA-treated group was observed when compared to the vitrified group (0.12 ± 0.03 vs. 4.17 ± 1.27, P=0.001) (Fig .2A). However, AA did not significantly affect the expression level of *Hdac1* (Fig .2B).

## Discussion

For the first time, in this study, AA -as a *Hat* inhibitor- was applied before vitrification of mouse oocytes. The results of this study proved that histone acetylation and *Hat* expression in vitrified mouse oocytes (M..) were elevated. Similarly, Spinaci et al. (13) and Suo et al. (11) showed that vitrification significantly increases the acetylation of histone H4 at lysine 12 and affects the expression of *Hat* in oocytes and zygotes derived from them. 

In this study, AA treatment before vitrification reduced AcH4K12 and *Hat* expression levels in comparison to those of oocytes without AA pretreatment. The inhibitory effect of AA on *Hats* was reported by Hemshekhar et al. (16), Sung et al. (21) and Yasutake et al. (22). 

AA obtained from the nutshell of *Anacardium occidentale* was used to design new *Hat* inhibitors (15). AA and its derivatives influence the transcription factor NF-KappaB (NF-.B) (16, 23).

It was demonstrated that the p50-p65 subunits, in their bounded inactive form, are linked to the inhibitor of KappaB (Iκαβ) protein in resting cells. Any stimulation can separate p50-p65 subunits from Iκαβ resulting in translocation of this dimer to the nucleus. By acetylation of p50-p65 subunits, its transcriptional ability and DNA- binding capacity are regulated. Hats are engaged with the target gene by these subunits (16, 21, 22). 

It has been claimed that vitrification induces either an increase in histone acetylation or a decrease in histone deacetylation, or both; thus, vitrification procedure may induce an imbalance between acetylation and deacetylation enzymes (13). 

This study showed that the level of *Hat1* in the vitrified MII oocytes was higher than that of the fresh control MII oocytes, while vitrified group oocytes showed decreased expression levels of *Hdac1*. 

It is well established that reductions in *Hdac1* lead to histone hyperacetylation. *Hdac1* is considered the main deacetylase in preimplantation embryos. A reduction in *Hdac1* expression leads to hyperacetylation of histone H4 at lysine 5 in development of preimplantation mouse embryos (24). 

Following AA treatment, the *Hat* expression was decreased in vitrified oocytes. The increase of histone acetyltransferase is perhaps due to vitrification, and AAcan inhibit this process.

In this study, we also demonstrated that H4K12 is analytical tools. All authors revised the manuscript acetylated during vitrification, whereas it is deacetylated and approved the final paper. in fresh control oocytes. In this study, there was a weak or no signal of acetylated H4K12 in MII oocytes in References the control group, which was also shown by previous studies (25). Changes in the acetylation state of H4K12 were observed immunocytochemically in the vitrified oocyte by using an antibody specific for acetylated H4K12. 

Incubating oocytes for 40 minutes with AA followed by vitrification, decreased the acetylation level of H4 during vitrification. This alteration was probably caused by AA which inhibits the enzymes responsible for histone acetylation. It was reported by Parthun (26) that *Hat1* probably induces the acetylation of the lysine 12 at histone 4. Indeed, existence of a balance between Hats, as the transcriptional coactivators, and Hdacs, which suppress transcription, is essential for the state of histone acetylation (27). 

Several studies have indicated that alterations in *Hats* and *Hdacs* affect epigenetics, and epigenetic changes may result in chromatin-modifying factors in many cancers (28). Furthermore, it was demonstrated that the increase in H4 acetylation levels was complemented by increasing *Hat* (29), as a significant association between the level of *Hat* expression and H4K12 acetylation was found.

It was observed that *Hat1* expression is elevated during vitrification, whereas *Hdac1* expression decreased during the cryopreservation procedure. These results were in agreement with those shown in the last studies (13). The results also indicated that the expression of *Hat1*, and the acetylation levels of lysine 12 residues on histones H4, were noticeably decreased by treating oocytes with AA, while no alteration was seen in *Hdac1* expression. In addition, the overall survival rate of oocytes after thawing was close to the findings of the study done by Cobo et al. (30). 

## Conclusion

The evidence from the present study suggests that AA pretreatment reduces *Hat* expression; also, our findings showed that acetylated state of H4K12 is decreased during vitrification. 
